# Come Now and Let Us Reason

**Published:** 1884-04

**Authors:** Thos. K. Beecher


### COME NOW AND LET US REASON.

THOS. K. BEECHER.

Reader! how many different ways can you invent for the disposition of the dead? So many different ways have already been not merely invented, but practiced; and in every case, when the fashion is once fixed, the people who practice that way of treating the dead, feel about it just as you do about burying—as if it were the only decent, reverent, pious way.

I will set up now, in the fewest and best words that I can command, all the ways of treating the dead that I have yet heard of.

Class I. *Various forms of Exposure!* as (1) Removal from among the living—mere desertion—with no care or thought as to what may happen to the body. (2.) Intelligent exposure on raised platforms, that the noxious fumes may escape, as smoke and gasses from tall chimneys, harming no one. (3.) Exposure as in India, on loftier iron gratings, that the birds may devour the dead. (4.) Exposure to drying winds as in Peru, until a harmless mummy results. (5.) Exposure to wild beasts, ants, and beetles, who in a very short time dispose of all offensive parts of the dead (6.) Not precisely an exposure, but very like it, the casting of the dead into rivers or the deep sea, to serve as food to alligators, fish, and other hungry aquatics.

Of these various modes and grades of exposure, perhaps the most poetic and least offensive is the practice of some maritime cities and peoples of sending monthly a dead ship far out to sea, and there leaving the dead in the dark unfathomed caves of ocean.

II. *Hiding or concealing the dead.* (1.) In natural caves, and crevices among the mountains; a usual proceeding in limestone lands, where caves are numerous. (2.) From caves the step is a short one to the excavation of artificial caves, and the erection of cave-like tombs:—like some of the ornamental cavelets in our Woodlawn [Elmira] as to which the visitor asks, "I wonder what that cost." (3.) The offensiveness of caves and tombs naturally calls for disinfectants and embalming. Thus came to pass the noble art and mystery of mummifying:—an art and mystery by which the highly civilized, and the teachers of the world six thousand years ago, have saved themselves up to serve as fuel for Egyptian fellahs or curiosities for Barnum's circus. (4.) The offensiveness of caves and tombs which led to one extreme, embalming, leads also to the other, burying; our own familiar method around whose obscenity we have adjusted many costly devices of piety, poetry, and art.

III. *Minor and eccentric ways:* as, composting the bodies; or burying them near to vines and trees. Other races of men, occasionally, eat their dead, as shipwrecked mariners have been known to do, and Californian pioneers round Donner lake. Some devote their dead to science and the dissecting table.

IV. *The burning of the dead.* There is not a land, nor an age, nor a race that has left a record in history, where the burning of the dead has not been practised more or less generally, and through periods of time longer or shorter.

——Will the reader set up these various ways of disposing of the dead, and like an impartial juryman inspect them and ask himself two questions: (1), what is the end or aim that they all have in common? and (2), which one of these methods accomplishes that end or aim most thoroughly, with least shock to the intelligent and thoughtful, and greatest suggestion of comfort and hope?

Set up these ways, I say, gentlemen of the jury! fair-minded readers that you are! They have all been fashionable in their day and place. They have all had thousands and millions of tear-blind mourners practising them. They have all been glorified with religious and poetic usages and associations. Set them up, I say, and answer the two questions above, which I now repeat in their most condensed form: (1), what end in common have all these methods? (2), which one of them compasses this end the most satisfactorily?

For myself I reply, all men everywhere must do something with their dead, because of their offensiveness, their decay. To get rid of this offence is the one end which all these methods have in common; and (2) this end which they all have in common is most quickly, thoroughly, poetically and even religiously accomplished by cremation, incineration, burning.

Why, then, do we not practice this method? To which the answer is that nothing is so difficult to overcome as an opinion or a usage that has no foundation in reason, nor justification of any kind. A mere habit of thought or conduct, a mere social usage of which you can say nothing whatever except "*they all do so*," is well nigh invulnerable and ineradicable; therefore, and therefore only, we go on burying our dead;—a practice grievous, insulting, and disgusting to a vivid thinker, approach it upon what side he may. Who dares think truly and habitually of the contents of the rosewood coffin, six feet under ground, containing the unspeakably loathsome remains of what was once a loved form? Remains struggling with all the might of their inanimate helplessness to get back to the clean dust and the pure air whence the mighty power of God once called them for the uses of life, but which are at present shut in by boards and glass and brick and stone and sand and clay, condemned to fester there for ten and sometimes a hundred years.

Or again in this valley I can take the enquirer to twenty-five or thirty graves, any one of which is charged with contagious malaria of frightful plague—charged to that degree that a subsoil plow will reach down and explode the infernal machine. If it were proposed to plant packages of dynamite, properly *capped*, here and there all over the land, and leave them for posterity to stumble on and take the consequences, there would be an instant uproar; these dynamiters and their business would be promptly arrested. But this is precisely what we are doing when we bury our dead. There is a street in London—Little Marlboro—in which they dare not excavate or dig for gas or water or sewer purposes, because the plague is buried there; and there is not in all this valley one single grave one year old that can be safely opened and left open.

Again, all our water springs and well heads are, as we know, fed by the sweet rain from heaven. This rain, filtering through the earth, comes to us cool and clear, and, with few excep tions, wholesome; therefore let us forthwith take all our poisons, all our diseases, all our decays and natural offences and with them prime this wide-lying filter of our drinking water. Oh, beautiful burial!

It is of record among those maritime races (and therefore fond of fish) who were wont to toss their dead into deep water, that by and by they lost their appetite for fish, we think with reason, and ceased to dispose of their dead in that way, and adopted some other. In like manner it would seem as if we might lose our relish for well-water and quit burying our dead and do something else with them.

For now, a third and final question. Of all the ways, gentlemen of the jury, of disposing of the dead which I have mentioned above, against which one do you say the greater number of forcible, even resistless objections lie? Our answer is, of all the methods ever practiced by men, burial can be least defended. Any other, every other method of treating our dead is better than the one we practice. We cannot change for the worse if we try. Therefore let us go to the opposite extreme, and build a chapel with a crematory attached, and be the slaves of fashion no longer, but rather the children of reason—and when we say, dust to dust, *ashes to ashes*, mean what we say.

---

Cremation and the growth of public sentiment in its favor is the subject of an editorial in the *Philadelphia Medical Times.* It is believed evident that a great change is taking place in public opinion in Europe, as well as this country, regarding it, burning the dead being no longer regarded as being so revolting or unnatural; and it even appears in the public papers as an item of news unworthy of special comment. It was unanimously advocated by the late meeting of the Congress of Hygiene at Geneva and governments called upon to remove legal restrictions, which in both England and France exist, although cremation societies, supported by prominent men, exist in both countries, and their efforts in this direction promise success. As its advocates get stronger opposition seems to be dying out. It is beginning to be understood that the two processes of disposal of the dead differ essentially only in time, the process of oxidization being accomplished by the one in a few hours, the other requiring a century. The objections have been principally sentimental, the strongest real argument being that it destroys evidence of crime in cases necessarily extremely rare where exhumation might otherwise be resorted to, and which might be met by requiring in some or all cases that bodies be kept several months prior to cremation. Our crowded surburban cemeteries are a constant menace to the public health, by poisoning the air and water, and act as centres for the spread of contagious disease. A cremation society and a creamatory furnace, it is believed, might be formed and sustained in the present state of public sentiment in Philadelphia. It is suggested that body-snatching need be no longer dreaded, an argument that would find much weight in the popular mind of certain localities.

---

### Malaria not a Disease of Low Lands Only.

Dr. A.C. Heffenger, of the U. S. Navy, writes in the Boston *Medical and Surgical Journal:* I have seen intermittent and remittent fevers as prevalent on the dry, sandy plains and through the mountains, *even up to the snow line*, of Peru, as I have ever seen them in Panama.

During the construction of the Oroyo railway from Lima to its present terminus, fifteen miles from the summit of the Cordilleras, a violent malarial fever broke out as soon as the ground was disturbed, which carried off several thousand workmen. This fever raged chiefly along the *rainless portions of the road, where the earth is always exceedingly dry and the soil thin,* though it extended to an elevation of *thirteen thousand feet, or nearly to the snow level.* A description of this epidemic, under the title of Oroyo fever, was published some years ago by Medical Director Browne, of the navy, and Dr. Ward, a surgeon on the road at the time.

Two years ago a detachment of troops from the Chilian army of occupation in Peru was sent up the Oroyo railway and across the Cordilleras; but no sooner had they reached the *snow line* than a malignant *malarial fever,* much resembling the Oroyo, appeared among them and fairly decimated the force. At the present time the Chilian troops quartered on the dry plains are suffering heavily from various forms of malarial fevers, though one not acquainted with the peculiarities of the country would pronounce their atmospheric and telluric surroundings unqualifiedly good.

